# Systematic profiling of alternative splicing in *Helicobacter pylori*-negative gastric cancer and their clinical significance

**DOI:** 10.1186/s12935-020-01368-8

**Published:** 2020-06-30

**Authors:** Chuan Liu, Chuan Hu, Zhi Li, Jing Feng, Jiale Huang, Bowen Yang, Ti Wen

**Affiliations:** 1 Department of Medical Oncology, the First Hospital of China Medical University, Shenyang, 110001 Liaoning China; 2 Key Laboratory of Anticancer Drugs and Biotherapy of Liaoning Province, the First Hospital of China Medical University, Shenyang, 110001 Liaoning China; 3 Liaoning Province Clinical Research Center for Cancer, Shenyang, 110001 Liaoning China; 4 Key Laboratory of Precision Diagnosis and Treatment of Gastrointestinal Tumors, Ministry of Education, Shenyang, 110001 Liaoning China; 5 Qingdao University Medical College, Qingdao, 266071 Shandong China

**Keywords:** Alternative splicing, *Helicobacter pylori*-negative gastric cancer, Immune cells, Prognosis

## Abstract

**Background:**

Alternative splicing (AS) may cause structural and functional variations in the protein to promote the proliferation of tumor cells. However, there is no comprehensive analysis of the clinical significance of AS in *Helicobacter pylori*-negative gastric cancer (HP^**−**^ GC).

**Methods:**

The clinical, gene expression profile data and AS events of 138 HP^**−**^ GC patients were obtained from the database named the cancer genome atlas. Differently expressed AS (DEAS) events were determined by a comparison of the PSI values between HP^**−**^ GC samples and adjacent normal samples. Unsupervised clustering analysis, proportional regression and Kaplan–Meier analysis were used to explore the association between clinical data and immune features and to establish two nomograms about the prognosis of HP^**−**^ GC. Finally, splicing networks were constructed using Cytoscape.

**Results:**

A total of 48141 AS events and 1041 DEAS events were found in HP^**−**^ GC. Various functions and pathways of DEAS events parent genes were enriched, such as cell-substrate junction, cell leading edge, focal adhension, and AMPK signaling. Seven overall survival (OS)-related and seven disease-free survival (DFS)-related AS events were used to construct the prognostic signatures. Based on the independent prognostic factors, two nomograms were established and showed excellent performance. Then, splicing regulatory networks among the correlations suggested that splicing factors were significantly associated with prognostic DEASs. Finally, the unsupervised clustering analysis revealed that DEAS-based clusters were associated with clinical characteristics, tumor microenvironment, tumor mutation burden, and immune features.

**Conclusion:**

Seven OS-related and seven DFS-related AS events have been found to be correlated with the prognosis of HP^**−**^ GC and can be used as prognostic factors to establish an effective nomogram.

## Background

Gastric cancer (GC) is the fifth most common malignancy and the third leading cause of cancer-related death worldwide [1]. Among many carcinogens of GC, *Helicobacter pylori* (HP) infection is one of the most important factors [2]. And according to The proportion of HP-positive gastric cancer (HP+ GC) is significantly higher than that of HP-negative gastric cancer (HP^−^ GC) patients, and over 90% of patients with GC have been infected with HP [3]. Several studies showed significant differences in histology and prognosis between HP+GC patients and HP^−^ GC patients [4–6]. Marrelli et al [5] concluded that HP^−^ GC had a more advanced stage and a more advanced pT classification than HP+ GC. And this result was consistent with the conclusion of another study, which illustrated that HP^−^ GC patients are in stage IV more and have worse overall survival (OS) compared to HP+ GC patients [6]. Although the prognostic biomarkers of HP+ GC have been widely studied previously [7–9], the related research is few in HP^−^ GC. Therefore, it is necessary for us to explore the prognostic factors of HP^−^ GC patients.

In recent years, alternative splicing (AS) has received widespread attention for its abnormal forms may cause structural and functional variations in the protein, therefore it could benefit growth and survival for the tumors [10, 11]. Many studies have clarified the role of AS events as prognostic factors in cancers, such as hepatocellular carcinoma [12] and colorectal cancer [13]. However, the effect of AS events on the prognosis of patients with HP^−^ GC is still unknown. In addition, as the relationship between GC and immune mechanisms is revealed [14] and AS events can also affect tumor immunity [15], we are encouraged to further understand the correlation between AS events and immune features in HP^−^ GC.

In this study, AS events data from the TCGA SpliceSeq data portal were used to identify the HP^−^ GC -related AS events, and we comprehensively analyze the prognostic potential of AS events on HP^−^ GC patients. Furthermore, we investigated the biological and immunological functions associated with these AS events to explore their relevant mechanisms.

## Methods

### Data acquisition and selection

The clinical data of HP^−^ GC patients and the corresponding gene expression profile data were obtained from the cancer genome atlas (TCGA). And the inclusion criteria were as follows:(1) Histological diagnosis of HP^−^ GC; (2) With complete data of RNA sequencing; (3) With complete clinical data, including gender, age, and AJCC TNM staging. In addition, patients with OS less than 30 days were excluded from the present study. Meanwhile, data of seven types AS events were collected from the TCGA SpliceSeq database [16], including alternate acceptor site (AA), alternate promoter (AP), alternate donor site (AD), alternate terminator (AT), exon skip (ES), mutually exclusive exons (ME), and retained intron (RI). The percent spliced in (PSI) value, which ranges from 0 to 1, was used to quantify the AS events. To generate reliable AS events, the filter condition was set (percentage of samples with PSI values ≥ 75, an average of PSI values ≥ 0.05). Finally, the Upset plot generated by the UpSetR package was used to illustrate the interactive sets between seven types of AS events [17].

### Identification of tumor-associated AS events and enrichment analysis

Directly comparing the biomarkers at different pathological state to screen hub biomarkers has been widely performed to determine tumor-associated biomarkers in past researches [13, 15]. Therefore, to determine the differential expression AS (DEAS) events, a comparison of the PSI values was made between the HP^−^ GC samples and the adjacent normal samples. AS events with adjusted P < 0.05 and |LogFC|>1.5 were defined as DEAS events. The parent genes of identified DEAS events were then incorporated into the enrichment analysis, including Gene Ontology (GO) and Kyoto Encyclopedia of Genes and Genomes (KEGG) pathways with metascape [18].

### Construction of the prediction model based on the DEAS

To further understand the role of DEAS events in HP^−^ GC patients, 138 patients with OS data were enrolled to study the OS-related DEAS events, and 116 patients with DFS data were selected to study the DFS-related DEAS events. Afterward, a univariate Cox proportional hazard model was performed to identify the prognostic DEAS events, including OS-related and DFS-related AS events. Then, the least absolute shrinkage and selection operator (LASSO) regression analysis was used to avoid overfitting [19]. After selecting the optimal OS-related and DFS-related AS events in the LASSO analysis, we further established two prognostic signatures based on the multivariate Cox proportional hazard model.

The risk scores of each patient were determined by the following formula:

$${\text{Risk score}}\; = \;\mathop \sum \limits_{i}^{n} PSI i*\beta i$$

β was defined as the regression coefficient.

The optimal cut-off value of the risk score was identified by the X-tile software [20]. Then, all patients were stratified into the low-risk group, moderate-risk group, and high-risk group. In order to compare the OS and DFS between the three groups, Kaplan–Meier survival analysis with log-rank test was performed. In addition, CIBERSORT package was used to quantify the 22 immune cells in all tumor samples, and samples with CIBERSORT P < 0.05 were enrolled in the analysis between risk score and immune cells. The correlation between risk score and immune cells was determined by the Spearman correlation test.

### Development of nomogram based on the DEAS events and clinicopathological data

To establish the nomogram for HP^−^ GC patients, the prognostic predictors were determined by univariate Cox proportional hazard model. Then, the predictors with a P-value less than 0.1 in the univariate analysis were selected into the multivariate Cox analysis, and the independent prognostic predictors were determined. Afterwards, two nomograms of OS and DFS were established by the “rms” package and C-index was selected to show the discrimination [21]. Moreover, calibration curves were generated to show the calibration of the nomogram. Finally, to investigate the clinical value of nomograms, the decision curve analysis (DCA) was performed [22, 23].

### Construction of splicing related network

The splicing factors (SFs) data were downloaded from the SpliceAid2 database. After that, the expression of 71 SFs was obtained from the TCGA data portal. Then, the differential expression analysis between tumor samples and normal samples was performed to determine the tumor-related SF. The Spearman test was performed to explore the relationship between tumor-related SFs and DEASs. It was considered as a significant association when r is more than 0.5 and p is less than 0.05. In addition, the interaction networks of DEASs and SFs were constructed using Cytoscape (3.7.2).

### Evaluation of correlation between DEAS events and clinical data, tumor microenvironment, tumor mutation burden, and immune features

After DEASs were identified, the classification of HP^−^ GC cohort was performed by the unsupervised consensus approach by the “Consensus Cluster Plus” package [24, 25]. According to the results, the optimal number of clusters could be identified and patients were divided into several clusters. Meanwhile, the ESTIMATE algorithm was performed to quantify the tumor microenvironment, including immune score and stromal score. Then, Wilcoxon test analysis was performed to compare the 22 types of immune cells, immune score and stromal score between clusters. Besides, the associations between clusters and clinicopathological variables, including AJCC TNM staging, histologic grade, age, and gender were also analyzed.

## Results

### Overview of AS events and identification of HP^−^ GC -related AS events

**Fig. 1:**
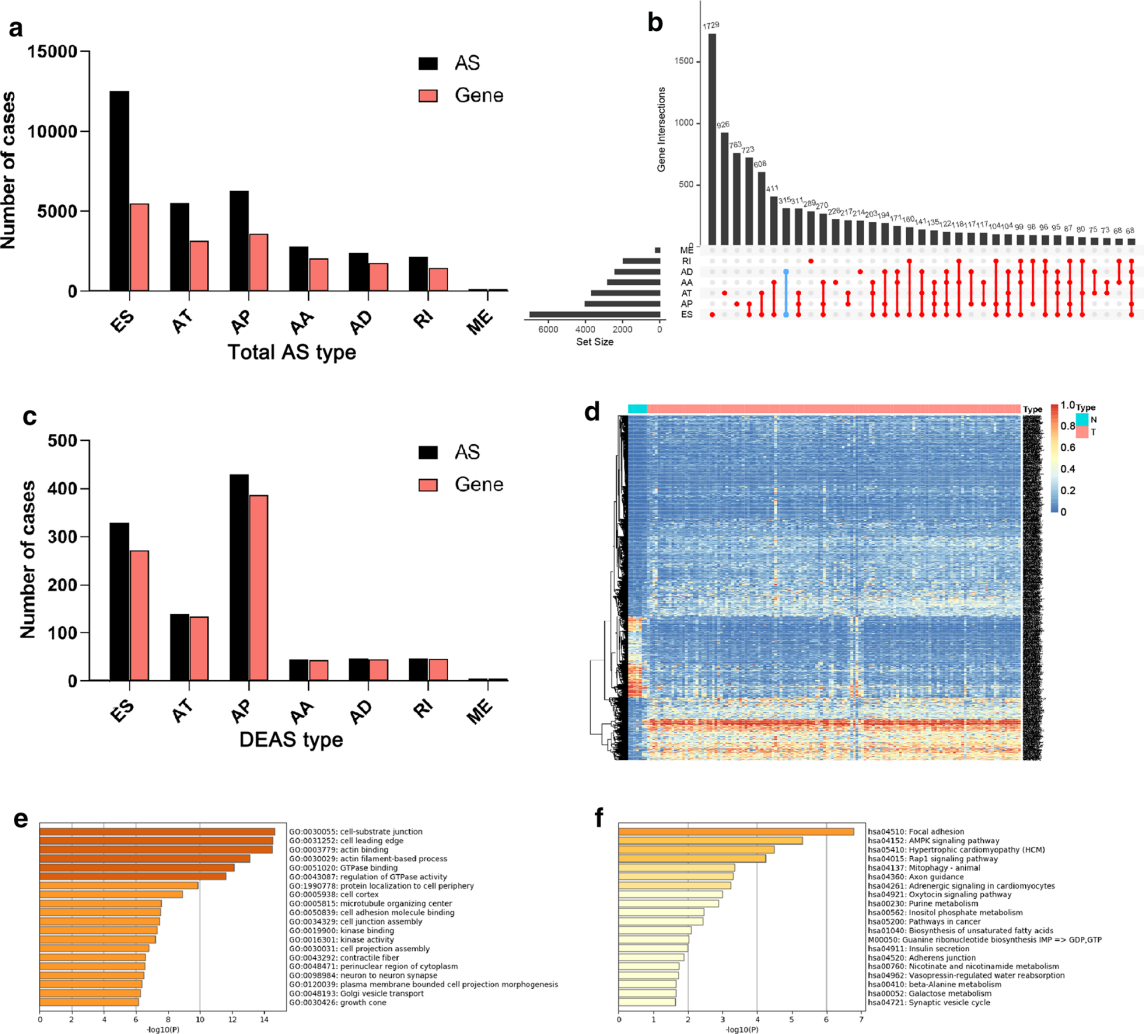
Overview of AS events and DEAS events and GO and KEGG enrichment analysis of parent genes in HP^**−**^ GC. **a** The number of AS events and parent genes in HP^**−**^ GC. **b** The Upset plot of interactions between seven types of AS events in HP^**−**^ GC. **c** The number of DEAS events and parent genes in HP^**−**^ GC. **d** The heat map shows the difference of PSI value of AS events between normal samples and HP^**−**^ GC; **e**, **f** GO and KEGG enrichment analysis of parent genes from DEAS events. *DEAS* differentially expressed alternative splicing, *HP*^***−***^*GC Helicobacter pylori*-negative gastric cancer, *GO* gene ontology, *KEGG* Kyoto Encyclopedia of Genes and Genomes pathways

Based on the criteria, 138 HP^−^ GC patients were included in our research. The characteristics and clinical data of the 138 patients were shown in Table 1. 31804 AS events were identified in 138 patients, including 2791 AA-type AS events containing 2084 genes, 2394 AD-type AS events involving 1767 genes, 6281 AP-type AS events including 3602 genes, 5530 AT-type AS events involving 3157 genes, 12524 ES-type AS events containing 5506 genes, 146 ME-type AS events involving 141 genes, 2138 RI-type of variable splicing event containing 1478 genes (Fig. 1a). Then, an Upset plot was generated to show the interactive sets of seven types of AS events. There are more than one AS events in most of the parent genes, and some single gene could have up to five distinct splicing patterns (Fig. 1b). For example, the blue line in the figure includes two points (AD and ES) means that there are 267 genes with only AD and ES.

**Table 1:** Clinicopathologic characteristics of patients with HP^**−**^ GC

Characteristics	Whole cohort (n = 138)
Gender	
Male	95
Female	43
Age	
< 65	51
≥ 65	87
T stage	
T1–2	38
T3–4	100
N stage	
N0–1	66
N2–3	72
M stage	
M0	131
M1	7
AJCC	
I–II	55
III–IV	83
Histologic grade	
G1	4
G2	59
G3	72
Gx	3

*HP- GC* Helicobacter pylori-negative gastric cancer

To identify the HP^−^ GC-specific AS events, differences of AS events between 138 primary HP^−^ GC tissue and seven adjacent normal tissues were compared to identify the DEASs profiling. Totally, 1041 DEASs were determined, which include 429 APs, 329 ESs, 139 ATs, 47 RIs, 47 ADs, 45 AAs and 5 MEs (Fig. 1c, d, Additional file 1: Table S1). Although most of the events in the HP^−^ GC cohort were ES events, the AP accounted for the largest part of DEASs, which indicated important roles of AP in HP^−^ GC patients and different roles of DEASs in cancer development.

### Enrichment and interaction analysis of DEAS

AS events could affect the protein function by various mechanisms [26]. Therefore, we should further understand the function of DEAS events in HP^−^ GC by studying the molecular mechanisms and pathways involved in the parent genes of DEAS events. The results of GO analysis were illustrated in Fig. 1e, which showed that specific GO categories were significantly related to HP^−^ GC, like cell-substrate junction, cell leading edge, actin binding, and actin filament-based process. In addition, some KEGG pathways that related to HP^−^ GC development were enriched (Fig. 1f), including focal adhension, AMPK signaling, hypertrophic cardiomyopathy, and rap1 signaling pathway. In a word, the enrichment analysis suggested corresponding genes of DEASs play an essential role in HP^−^ GC, which helped reveal the potential modification mechanisms of protein function by DEAS events.

### Development of the prognostic model for HP^−^ GC

**Fig. 2:**
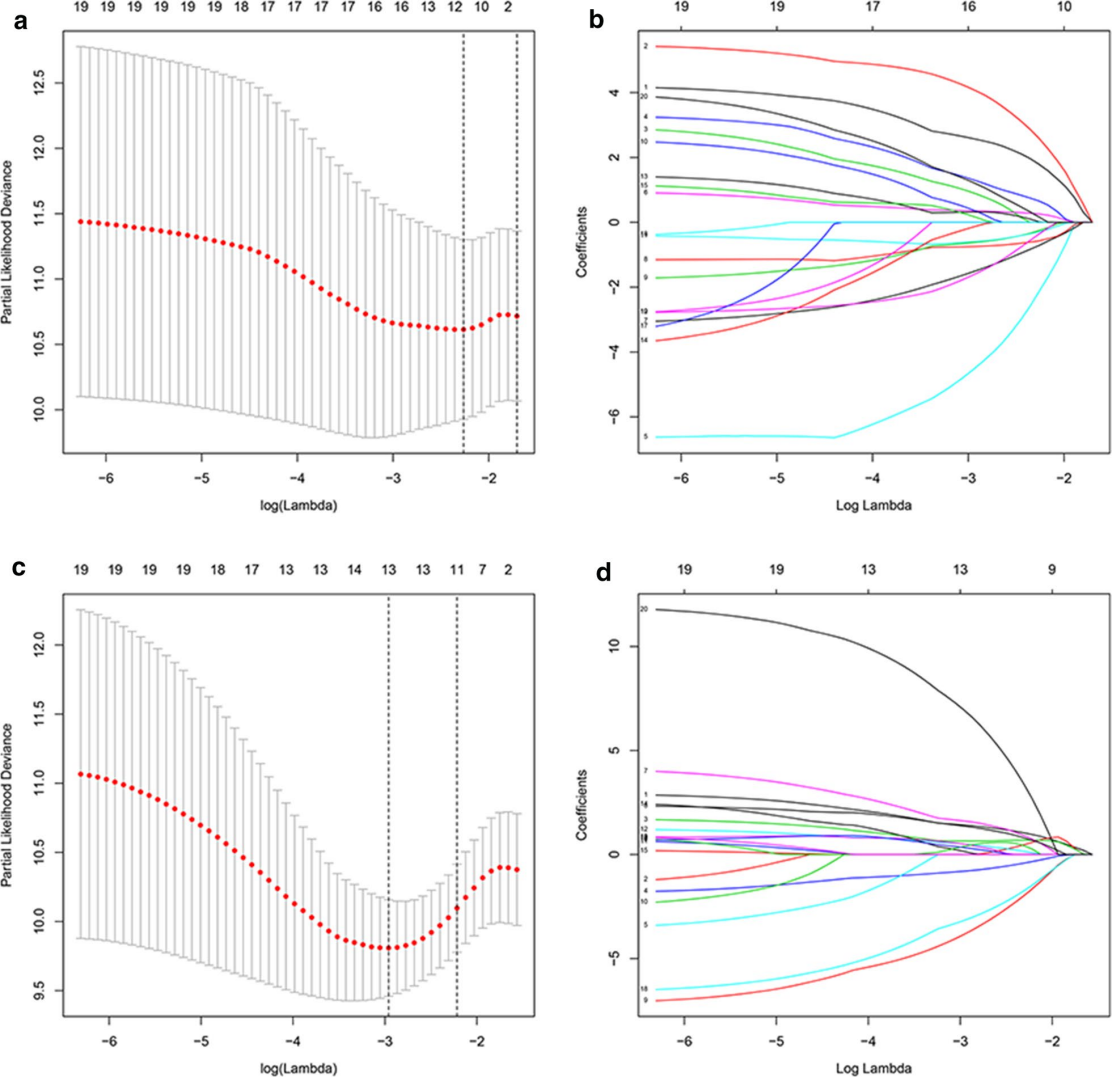
LASSO regression to select the significant OS-related DEAS events (**a**, **b**) and DFS-related DEAS events (**c**, **d**), respectively. LASSO: least absolute shrinkage and selection operator. *AS* alternative splicing, *OS* overall survival, *DFS* disease-free survival

Firstly, a univariate Cox proportional hazard model was conducted to determine the prognostic DEAS events, and 67 DEAS events were identified as the OS-related DEAS events, including 30 APs, 24 ESs, 6 ATs, 4 RIs, 2 ADs, and 1 AA (Additional file 2: Table S2). Meanwhile, 96 DEAS events were identified as the DFS-related DEAS events, which included 50 APs, 19 ESs, 8 ATs, 9 RIs, 5 ADs, 4 AAs, and 1 ME (Additional file 3: Table S3). Then, the LASSO regression was used to select the significant OS-related and DFS-related DEAS events (Fig. 2a–d), and 11 OS-related DEAS events and 13 DFS-related DEAS events were determined (Additional file 4: Table S4 and Additional file 5: Table S5). After the LASSO analysis, the significant DEAS events identified were analyzed by the multivariate Cox proportional hazard model and the prognostic models were established. Finally, seven OS-related DEASs and seven DFS-related DEASs events were determined as independent prognostic biomarkers (Tables 2, 3) in HP^−^ GC, which indicated that the DEAS events have both vital functions for biology and value for prognosis.

**Table 2:** OS-related DEAS events for HP^**−**^ GC patients

Gene	Type	ID	Coef	HR	95% CI	P value
TTC39C	AP	44852	4.203	66.893	7.273–615.256	0.000
WDPCP	AP	53726	7.319	1509.442	61.883–36818.171	0.000
ANKRD13A	AP	24394	− 8.225	0.000	0.000–0.403	0.028
BNIP3	AT	13478	− 3.641	0.026	0.001–0.671	0.028
APOD	ES	68181	− 1.594	0.203	0.024–1.701	0.142
KLC1	ES	29474	− 3.934	0.020	0.001–0.359	0.008
RELL1	AT	69003	3.072	21.593	1.335–349.130	0.030

*OS* overall survival, *DEAS* differently expressed alternative splicing, *HP*^***−***^*GC Helicobacter pylori*-negative gastric cancer, *AP* alternate promoter, *AT* alternate terminator, *ES* exon skip, *HR* hazard ratio, *CI* confidence interval

**Table 3:** DFS-related DEAS events for HP^**−**^ GC patients

Gene	Type	ID	Coef	HR	95% CI	P value
POLM	RI	79446	2.321	10.186	2.266–45.797	0.002
PLCD1	AP	64008	− 1.566	0.209	0.057–0.771	0.019
C9orf156	AP	87024	3.492	32.849	1.576–684.536	0.024
ELMOD3	RI	54213	2.629	13.856	1.331–144.215	0.028
TCF12	ES	30789	− 6.138	0.002	0.000–0.043	0.000
SYBU	AP	84909	− 4.891	0.008	0.000–0.179	0.002
ZFYVE21	ES	29513	11.239	76,054.320	322.567–17,931,942.782	0.000

*DFS* disease-free survival, *DEAS* differently expressed alternative splicing, *HP*^***−***^*GC Helicobacter pylori*-negative gastric cancer, *RI* retained intron, *AP* alternate promoter, *ES* exon skip, *HR* hazard ratio, *CI* confidence interval

**Fig. 3:**
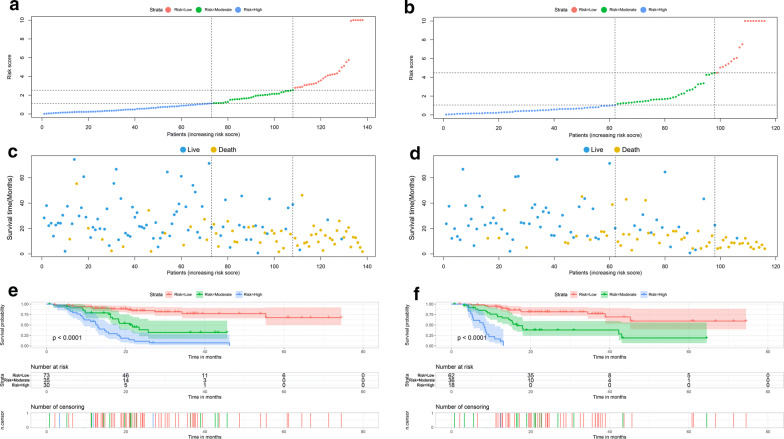
Establishment of the prognostic model for OS (**a**, **c**, **e**) and DFS (**b**, **d**, **f**) based on the independent DEAS events. (A, B) The risk curve of each sample based on risk score. **c**, **d** The scatter plot showed the survival status of HP^**−**^ GC patients. Kaplan–Meier survival curve of OS (**e**) and DFS (**f**) among three groups. *OS* overall survival, *DFS* disease-free survival, *AS* alternative splicing

Risk scores were calculated based on the selected DEAS events in the multivariate Cox analysis (risk score of OS signature = 4.203*TTC39C_44852_AP+ 7.319*WDPCP_53726_AP+ − 8.225*ANKRD13A_24394_AP+ − 3.641* BNIP3_13478_AT+ − 1.594* APOD_68181_ES+ -3.934*KLC1_29474_ES+ 3.072*RELL1_69003_AT; and risk score of DFS signature = 2.321* POLM_79446_RI + − 1.566* PLCD1_64008_AP+ 3.492* C9orf156_87024_AP+ 2.629* ELMOD3_54213_RI+ − 6.138* TCF12_30789_ES+ − 4.891* SYBU_84909_AP+ 11.239* ZFYVE21_29513_ES), and HP^−^ GC patients were stratified into low-, middle^−^ and high-risk groups through the X-tile software. The distribution of prognostic outcomes in these three risk stratifications was visualized in Fig. 3a, b. As shown in Fig. 3c, d, compared with the low^−^ risk group, the high-risk group had a significantly higher incidence of deaths and shorter survival time of patients. In addition, the K-M survival curves and the log-rank test showed that both of the prediction models had excellent performance for predicting the prognosis of HP^−^ GC patients in these three groups (Fig. 3e, f).

**Fig. 4:**
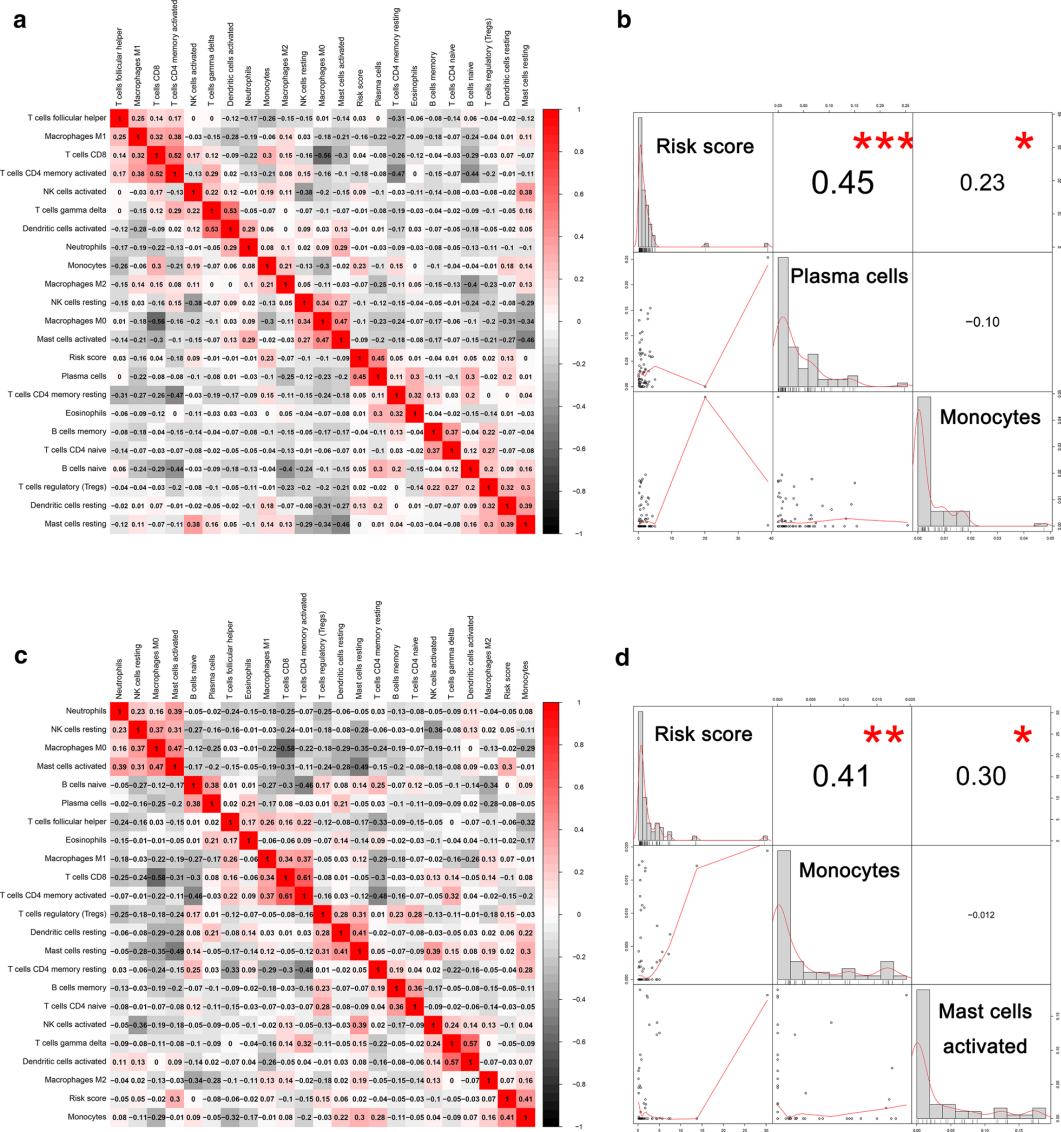
Association between 22 types of immune cells and risk score based on DEAS events. **a** The correlation between immune cells and the risk score of OS. **b** Correlation analysis of specific immune cells and risk score of OS. **c** The correlation between immune cells and risk score of DFS. **d** Correlation analysis of specific immune cells and risk score of DFS. *OS* overall survival, *DFS* disease-free survival, *DEAS* differentially expressed alternative splicing; *p<0.05; **p<0.01; ***p<0.001

The tumor immune mechanisms play essential roles in the progress of various cancers [27]. Thus, we evaluated the association between 22 types of immune cells and risk scores of OS and DFS to explore the correlation between immune cells and DEAS-based prognostic value in HP^−^ GC (Fig. 4a, c). It is showed that plasma cells (r = 0.45) and monocytes (r = 0.23) are associated with the risk score of OS, while monocytes (r = 0.41) and mast cells activated (r = 0.30) are related with a risk score of DFS (Fig. 4b, d).

### Development of AS-clinicopathologic nomogram

**Fig. 5:**
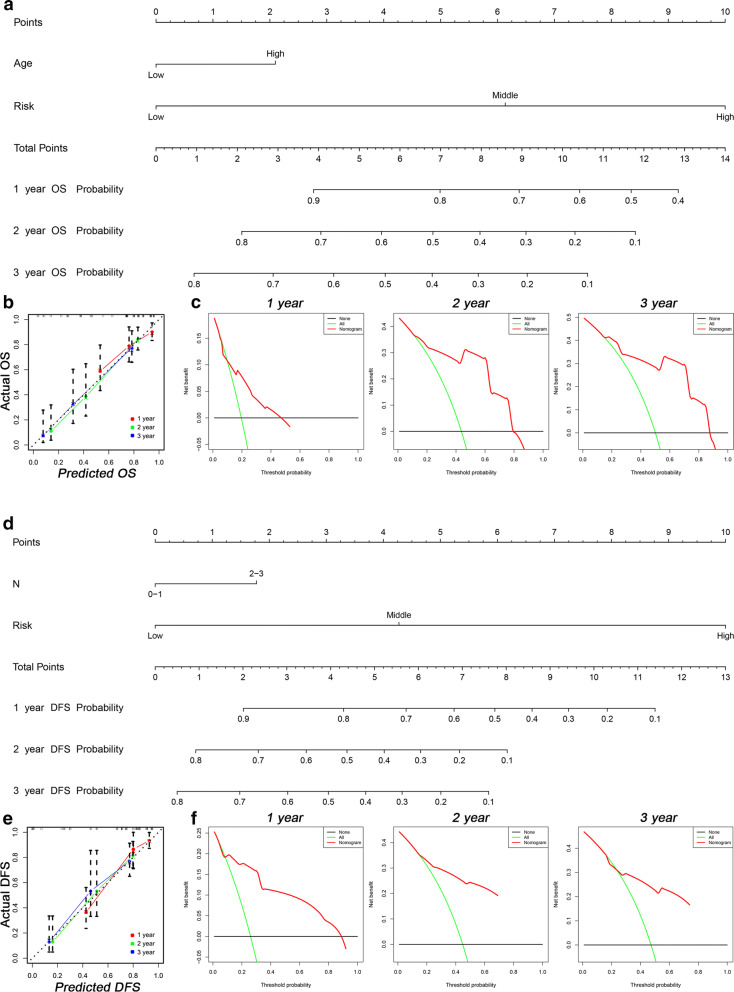
Two nomograms and corresponding results showed the prognostic value of AS events and clinicopathologic data. Nomogram for predicting OS (**a**) and DFS (**d**) in the HP^**−**^ GC cohort. Calibration plot of the AS-clinicopathologic nomogram in terms of the agreement between nomogram-predicted and observed 1-, 2-, and 3-years OS (**b**) and DFS (**e**) in the HP^**−**^ GC cohort. **c**, **f** Decision curve analysis of the AS-clinicopathologic nomogram for 1-, 2-, and 3-years risk in the HP^**−**^ GC cohort. *AS* alternative splicing, *OS* overall survival, *DFS* disease-free survival, *HP*^***−***^*GC Helicobacter pylori*-negative gastric cancer, *ROC* receiver operating characteristic

Nomogram is a tool used for the prediction of patients’ prognosis. The Cox analysis of clinicopathologic characteristics showed that in addition to the risk score was one of the independent factors for OS and DFS in HP^−^ GC cohort, age and N stage were OS-related and DFS-related factors, respectively (Tables 4, 5). Then, two nomograms were established for predicting the OS (Fig. 5a) and DFS (Fig. 5d) in HP^−^ GC patients. And the plot of the AS-clinicopathologic nomograms to predicting the possibility of survival at 1-, 2-, and 3-years showed a strong consistency between the nomogram-predicted outcome and actual outcome (Fig. 5b, e). The C-index for OS nomogram was 0.762 (95% CI 0.729 to 0.795), which was higher than risk score (0.740, 95% CI 0.708 to 0.772) and age (0.552, 95% CI 0.519–0.585). And the C-index for DFS nomogram was 0.783 (95% CI 0.750 to 0.816), which was higher than risk score (0.767, 95% CI 0.738 to 0.796) and T stage (0.554, 95% CI 0.518–0.590). The DCA of nomograms for 1-, 2-, and 3-years was also established (Fig. 5c, f). It is worth noting that the decision curves for 1 year, 2 years, and 3 years all showed higher credibility, which suggested the nomogram based on AS events and clinical data is effective for predicting the survival probability and prognosis of patients.

**Table 4:** Univariate Cox analysis of clinicopathologic characteristics

	OS			DFS		
	HR	95% CI	P value	HR	95% CI	P value
Age	1.854	1.040–3.305	0.036	2.02	0.775–5.266	0.150
Sex	1.714	0.939–3.131	0.079	2.67	1.299–5.488	0.008
T	1.864	0.913–3.803	0.087	1.364	0.713–2.608	0.348
M	1.362	0.492–3.776	0.552	0.735	0.175–3.09	0.674
N	1.999	1.160–3.445	0.013	1.597	0.916–2.783	0.099
AJCC	2.083	1.157–3.752	0.014	1.544	0.861–2.77	0.145
Grade (x)		1.040–3.305	0.504		0.775–5.266	0.993
Grade (1)	1.713	0.231–12.713	0.599	1.277	0.172–9.483	0.811
Grade (2)	2.469	0.337–18.112	0.374	1.326	0.180–9.779	0.782
Grade (3)	1.958	0.122–31.430	0.635	0.000	0.000–0.000	0.975
Low risk			0.000			0.000
Middle risk	4.442	2.122–9.299	0.000	4.027	2.018–8.038	0.000
High risk	11.614	5.783–23.323	0.000	24.974	10.726–58.147	0.000

*OS* overall survival, *DFS* disease-free survival, *HR* hazard ratio, *CI* confidence interval

**Table 5:** Multivariate Cox analysis of clinicopathologic characteristics

	OS			DFS		
	HR	95%CI	P value	HR	95%CI	P value
Low risk			0.000			0.000
Middle risk	0.292	0.188–0.455	0.000	3.910	1.953–7.828	0.000
High risk	1.169	0.790–1.728	0.435	22.745	9.479–54.577	0.000
Age	1.945	1.071–3.533	0.029			
Sex	1.089	0.574–2.066	0.795	1.682	0.790–3.582	0.178
T	1.039	0.440–2.457	0.930			
N	1.425	0.696–2.915	0.333	1.771	1.015–3.091	0.044
AJCC	1.468	0.6153.505	0.387			

*OS* overall survival, *DFS* disease-free survival, *HR*HR: Hazard ratio,*CI* Confidence interval

### Construction of AS-SFs network

**Fig. 6:**
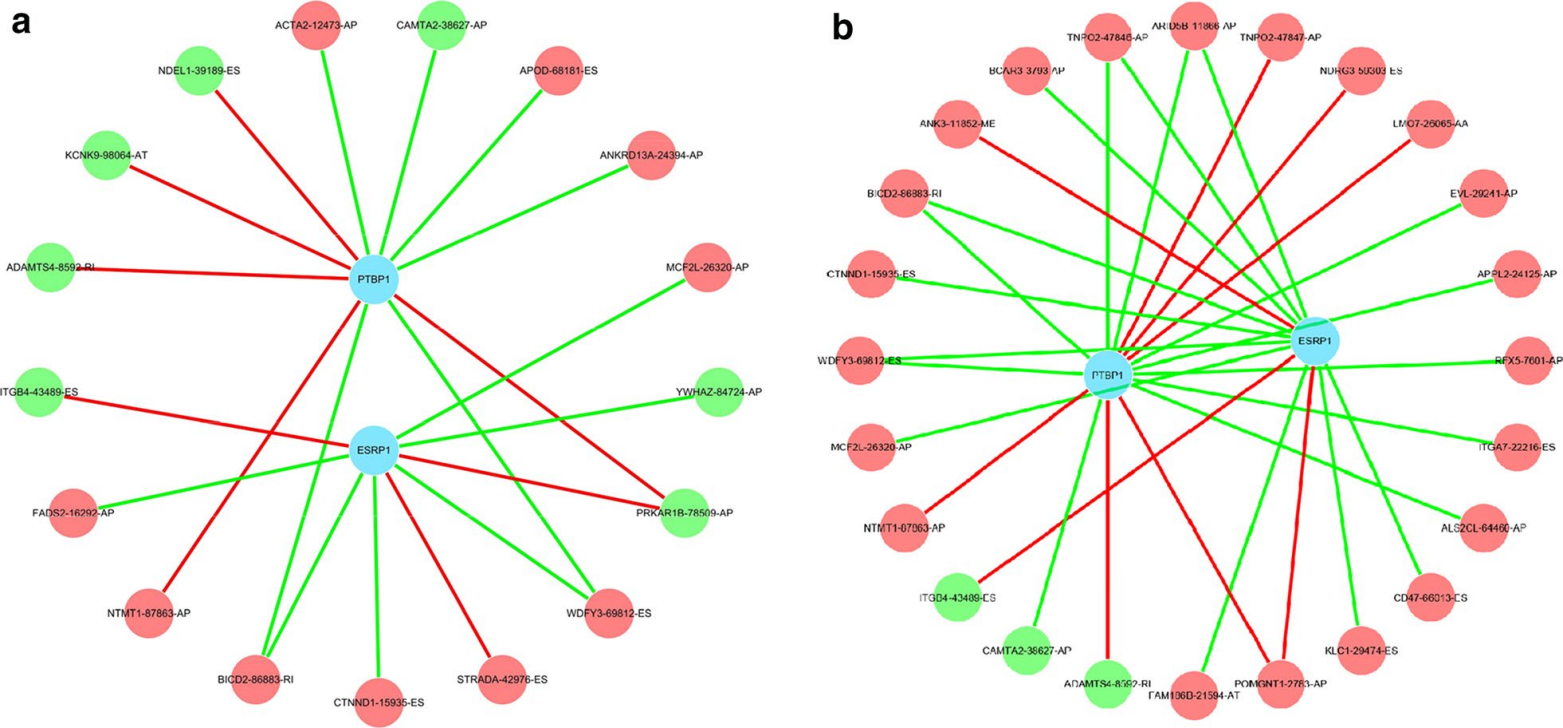
Correlation network between OS-AS events (**a**) and DFS-AS events (**b**) and prognostic SFs in HP^**−**^ GC. The majority of prognostic AS events with poor prognosis (red dots) were positively (green lines) correlated with the expression of SFs, while the majority of AS events with good prognosis (green dots) were negatively (red lines) correlated with the expression of SFs. AS: alternative splicing; OS: overall survival; DFS: disease-free survival; SF: splicing factor; HP^**−**^ GC: *Helicobacter pylori*-negative gastric cancer. HP^**−**^ GC: *Helicobacter pylori*-negative gastric cancer

SFs are elements for regulating AS events. Compared with the normal tissues, alterations of SFs in tumors promote differential splicing patterns, which lead to an increase of pro-tumorigenic isoforms [28]. Therefore, it’s important for us to understand whether DEAS events are regulated by specific key SFs in HP^−^ GC. In order to understand this issue, we firstly identified six tumor-related SFs through differential analysis (Additional file 6: Table S6). Then, we analyzed the relationship between the expression of these SFs and prognostic DEAS events, including OS-related DEAS events and DFS-related DEAS events. Two splicing regulatory networks were shown in Fig. 6. Each SF was significantly correlated with more than one OS-related and DFS-related DEAS event, reflecting the intricately cooperative and competitive association between SFs and AS events [29].

### AS-based clusters associated with clinical data, tumor microenvironment, tumor mutation burden and immune features

**Fig. 7:**
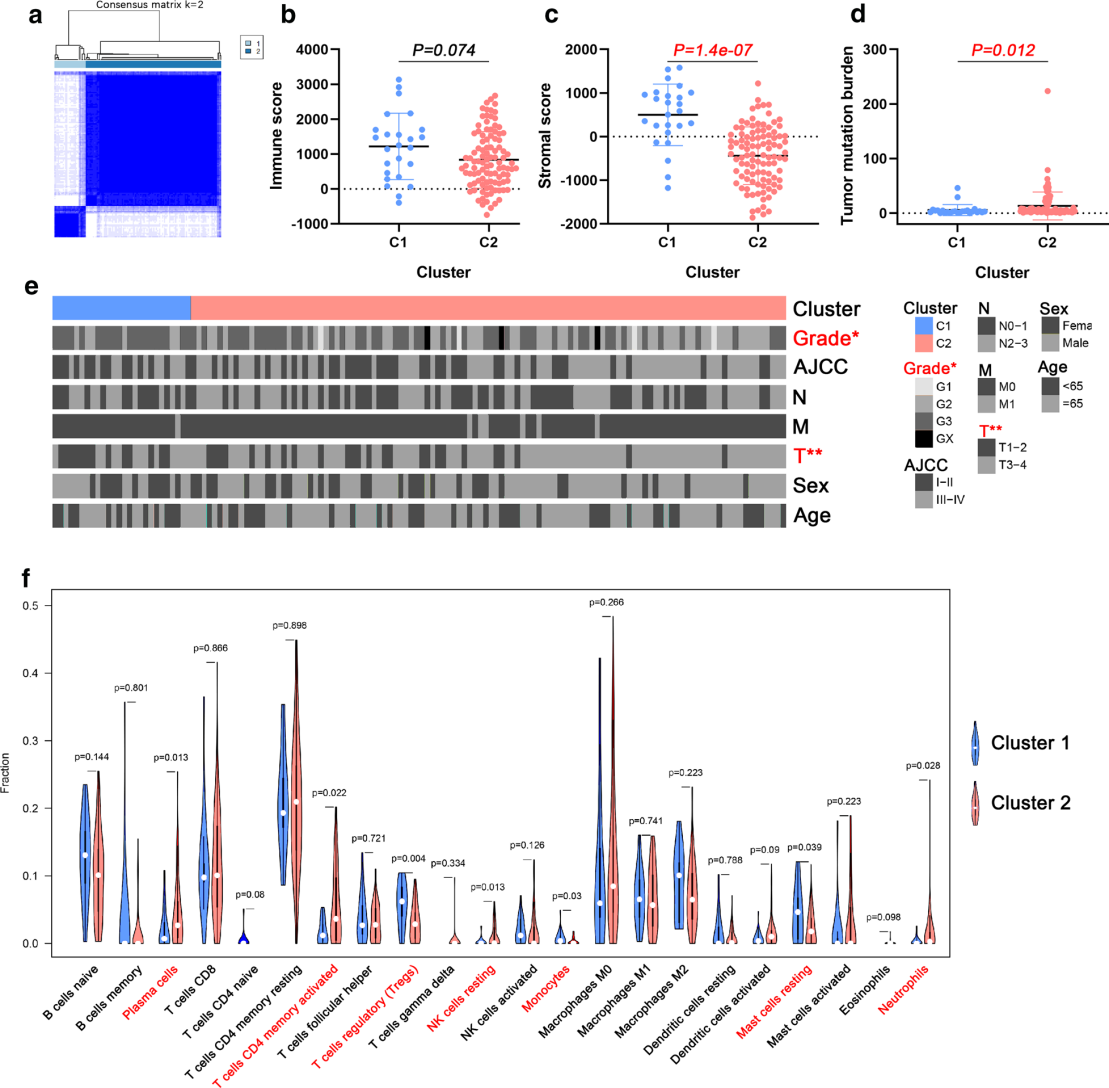
AS events-based clusters significantly associated with immune cells. **a** Consensus clustering analysis identification of two clusters (samples, n = 138). **b**, **c** Immune score and stromal score between AS-based clusters. **d** Tumor mutation burden between AS-based clusters. **e** Heat map of the DEAS events ordered by cluster, with annotations associated with each cluster. Chi square test was used. **f** Statistical differences in each type of immune cell between C1 and C2. *AS* alternative splicing, *DEAS* differentially expressed alternative splicing

Targeting at the individual level of cancer patients, the various expressions of each DEAS reflected the prognosis of some patients and can predict different clinical outcomes [15]. Therefore, we further identified the different AS patterns by unsupervised analysis based on the DEASs. By using the Elbow method and Gap statistic method to determine the optimal number of clusters, we finally determined the two clusters of samples: C1 (n = 26, 18.8%) and C2 (n = 112, 81.2%) (Fig. 7a).

In order to expound the clinicopathologic characteristics of the DEAS clusters, the association of clusters with clinal status was firstly explored and showed in a heat map (Fig. 7e). It revealed that the distribution of T stage and grade in HP^−^ GC samples between two clusters was not random. Then, by using the ESTIMATE algorithm, we calculated immune and stromal scores to perform quantitative analysis of the presence of stromal cells and immune cells in HP^−^ GC patients. As shown in Fig. 7b, c, the stromal score of C1 is significantly higher than that of C2. In addition, the result showed in Fig. 7d suggested that C2 had a significantly higher tumor mutation burden compared to C1. Meanwhile, by comparing the composition of 22 types of immune cells between the C1 (n = 17) and C2 (n=54), we found that there were significant differences between the two clusters in plasma cells, T cells CD4 memory resting, T cells regulatory (Tregs), NK cells resting, monocytes, mast cells resting and neutrophils (Fig. 7f).

## Discussion

GC is one of the cancers with the highest morbidity and mortality [30]. Finding effective prognostic and diagnostic markers for GC has become the focus of future research. However, because of the limitations of traditional gene identification methods, it has not brought obvious advantages to disease diagnosis and clinical results. Recently, with the development of gene sequencing technology, AS events have been widely studied and has shown some progression [31]. The biochemical mechanisms of AS events are complex and remain poorly understood to a large extent, but its importance for gene regulation cannot be ignored [32]. Abnormality of AS events have been shown to be associated with the occurrence, development and distant metastasis in many cancers [33]. Compared with a previous study reporting the relationship between GC and AS events [34], this study focused on the value of AS events in HP^−^ GC and visual display patients’ prognosis by nomograms. In addition, the correlation between DEASs and tumor microenvironment, immune features and tumor mutation burden was studied to provide the basis for HP^−^ GC immune and molecular mechanisms.

In the present study 1041 DEAS events from 930 genes were identified in HP^−^ GC. In the results, a series of molecular mechanisms and pathways were significantly enriched in these genes through GO and KEGG enrichment analyses. Several of the enriched molecular mechanisms have been shown to promote tumorigenesis. For example, GTPase activity has a promoting effect on the metastasis and invasion of prostate cancer cells [35]. AMP-activated protein kinase (AMPK) can regulate cellular energy metabolism, and stimulate ATP generation [36]. Activated AMPK promotes glycolysis and enhances cellular differentiation of tumors [37]. These analysis results provide a basis for HP^−^ GC’s molecular mechanisms of AS events and provide a basic theory for subsequent experimental verification. In addition, to explore the tumor-related mechanisms of total parent genes in DEAS events, the parent genes of independent prognostic AS events have been discovered. The gene of BNIP3 in the AS we found can promote hypoxic survival and autophagy of cancer cells [38]. And autoantibodies against proteins translated by the RELL1 gene are considered as underlying biomarkers for detecting early-stage breast cancer [39].

In order to further understand the prognostic value of AS events in HP^−^ GC, the seven OS-related and seven DFS-related prognostic AS events were included in the calculation of risk score to establish a prediction model. In previous studies, the median was often used as a cut-off value to divided patients into high-risk and low-risk groups. For the accuracy of the results, X-tile, a new and useful tool for bioinformatic analysis, was chosen to confirm the cut-point of the risk score. The survival status of three groups suggested that AS events can affect patient survival time and prognosis of HP^−^ GC patients. Besides studying the relationship between AS events and prognosis, we also include clinicopathological data of HP^−^ GC patients into this research to describe the prognosis more comprehensively.

According to the discovery of the important role of immune mechanisms in the GC progression in a previous study [14], the immune environment is considered as a key determinant of GC. Macrophages, neutrophils, dendritic cells, and immune cells of various T cell lineages are the major components of the tumor microenvironment and are involved in many processes of tumorigenesis and growth [40]. Besides, previous studies showed that AS events may be associated with immune cell infiltration by regulating tumor-associated immune cytolytic activity [41]. Therefore, in the AS events we determined, correlation analysis on 22 types of immune cells and risk scores was also be performed. From the results of the correlation coefficient, we can conclude that there was a strong correlation between monocytes and both DFS (r = 0.41) and OS (r = 0.23). Similarly, Zhang et al. [42] found that infiltrating immune cells were associated with survival, therapeutic responses and prognosis of breast cancer patients and monocytes were decreased in cancer patients with higher-grade tumors. From the perspective of clusters to further explore the association between AS events with clinical data, tumor mutation burden and immune features. It is obvious that the T stage of C2 and tumor mutation burden is higher than that of C1, while the stromal score is lower. It indicated that the tumor mutation burden may be positively associated with the T stage, while the stromal score may be negative with it. This conclusion was similar to the previous one that higher tumor mutation burden tends to achieve a prognosis and to promote the infiltrations of immune cells such as T cells and NK cells in bladder cancer [43]. Pan et al proved that the infiltration of immune cells is closely related to tumor progression and prognosis [44]. The reason that there is no difference in immune score between C1 and C2 in our study may be the small sample size. But in fact, the infiltration of immune cells being involved in the evolution of GC has been clarified [45, 46]. From the comparison results of the two groups in 22 types of immune cells, it can be seen that there are differences in the composition ratios of some immune cells, indicating that immune features are significantly associated with AS events. Different gene splicing patterns can be affected by antigen stimulation to regulate immune cell activation thresholds and to maintain internal environment stability [47]. The type I membrane protein receptor carcinoembryonic antigen-related cell adhesion molecule 1 (CEACAM1) differently exhibits significant AS events and is highly expressed in various types of immune and parenchymal cell [48]. It can act on immunity such as inhibiting natural killer cell-mediated cytotoxicity, regulate neutrophil and monocyte development and function [48]. Besides, it also regulates T cell activation and mediates tolerance [49]. There are various splicing forms of PyTEPs in Yesso scallo, of which involvement participates in the immune response through different response models [50]. Various studies have shown that AS events are related to the distribution of immune cells, which were consistent with the conclusions of this study.

SFs can recognize and combine pre-mRNA codon-regulated genes, and then influence the selection of exons and the choice of splice sites to achieve the purpose of regulating AS events [51]. Therefore, we comprehensively analyzed SFs and its expression to elucidate the splicing mechanism of HP^−^ GC. The abnormal expression conditions of epithelial splicing regulatory protein 1 (ESRP1) and polypyrimidine tract binding protein 1 (PTBP1) are closely related to the expression of AS events. ESRP1 is one of the earliest epithelial restriction RNA binding proteins discovered, which can regulate AS of multiple epithelial transcripts [52]. Establishing the network between OS-related AS events and tumor-specific SFs, this research found that ESRP1 was associated with several OS-AS events that were correlated with OS. Similarly, ESRP1 was considered avital SF that leads to the progression and metastasis in pancreatic and prostate cancer [53, 54]. While in addition to its role in splicing, PTBP1 also participates in the regulation of other aspects of RNA metabolism. Previous studies have shown that PTBP1 suppresses cell viability and promotes apoptosis during lung tumorigenesis [55].

This study still has some limitations. Firstly, it was a retrospective study, of which predictive models were on the basis of public databases. So, data from other regions are not included. Second, owing to the incidence of the disease was relatively low and the sample size included was small, the predictive power of some results needs to be increased and experimentally verify the role of AS events in HP^−^ GC by enlarging the sample size. Finally, there is less immune information, including only the situation of immune cells. In order to fully explore the immune mechanisms associated with HP^−^ GC, more immune information should be included, such as immunohistochemistry results and immune checkpoint detection.

## Conclusion

In summary, the present study showed the prognostic value of AS events which play important roles in HP^−^ GC tumorigenesis. Besides, our research also suggested that some prognostic SFs may be related to potential mechanisms of the splicing forms by regulating AS events. More importantly, the risk^−^ classification based on AS events is not only crucial for deciphering tumorigenesis mechanisms, but also reveals the correlation between molecular changes and immune characteristics, which can be used as underlying prognostic biomarkers and therapeutic targets for HP^−^ GC patients.

## Supplementary information

**Additional file 1: Table S1.** 1041 DEASs determined by comparing HP^**-**^ GC to adjacent normal tissues.

**Additional file 2: Table S2.** 67 OS-related DEAS events determined by univariate Cox regression.

**Additional file 3: Table S3.** 96 DFS-related DEAS events determined by univariate Cox regression.

**Additional file 4: Table S4.** 11 OS-related DEAS events determined by LASSO regression.

**Additional file 5: Table S5.** 13 DFS-related DEAS events determined by LASSO regression.

**Additional file 6: Table S6.** Six tumor-related SFs determined by differential analysis.

## Data Availability

The data of this study are from the TCGA database.

## Abbreviations

AS: Alternative splicing
HP**–**GC: *Helicobacter pylori*-negative gastric cancer
TCGA: The cancer genome atlas
DEAS: Differently expressed alternative splicing
OS: Overall survival
DFS: Disease-free survival
SF: Splicing factor
GC: Gastric cancer
HP+ GC: *Helicobacter pylori*-positive gastric cancer
AA: Alternate acceptor site
AP: Alternate promoter
AD: Alternate donor site
AT: Alternate terminator
ES: Exon skip
ME: Mutually exclusive exons
RI: Retained intron
PSI: Percent spliced in
GO: Gene ontology
KEGG: Kyoto Encyclopedia of Genes and Genomes
LASSO: The least absolute shrinkage and selection operator
DCA: Decision curve analysis
AMPK: AMP-activated protein kinase
CEACAM1: The type I membrane protein receptor carcinoembryonic antigen-related cell adhesion molecule 1
ESRP1: Epithelial splicing regulatory protein 1
PTBP1: Polypyrimidine tract binding protein 1

## References

[1] Bray F, Ferlay J, Soerjomataram I, Siegel RL, Torre LA, Jemal A (2018). Global cancer statistics 2018: GLOBOCAN estimates of incidence and mortality worldwide for 36 cancers in 185 countries. CA Cancer J Clin.

[2] Plummer M, Franceschi S, Vignat J, Forman D, de Martel C (2015). Global burden of gastric cancer attributable to *Helicobacter pylori*. Int J Cancer.

[3] Yoon H, Kim N, Lee HS, Shin CM, Park YS, Lee DH, Jung HC, Song IS (2011). *Helicobacter pylori*-negative gastric cancer in South Korea: incidence and clinicopathologic characteristics. Helicobacter..

[4] Alfarouk KO, Adil HHB, Ahmed NA, AbdelRahman MR, Abdel KM, Sari TSA, Abdelhamid H, Gamal OE, Muntaser EI, Saad SA, Shakir DA, Claudiu TS, Cyril R, Rosa AC, Stephan JR, Stefano F, Salvador H (2019). *Helicobacter pylori* the possible role of in gastric cancer and its management. Front Oncol.

[5] Wroblewski LE, Peek RM, Wilson KT (2009). Negative *Helicobacter pylori* status is associated with poor prognosis in patients with gastric cancer. Cancer.

[6] Tsai KF, Liou JM, Chen MJ, Chen CC, Kuo SH, Lai IR, Yeh KH, Lin MT, Wang HP, Cheng AL, Lin JT (2017). Distinct clinicopathological features and prognosis of *Helicobacter pylori* negative gastric cancer. PLoS ONE.

[7] Ranjbar R, Hesari A, Ghasemi F, Sahebkar A (2018). Expression of microRNAs and IRAK1 pathway genes are altered in gastric cancer patients with *Helicobacter pylori* infection. J Cell Biochem.

[8] Lin S, Zhang Y, Hu Y, Yang B, Cui J, Huang J, Wang JM, Xing R, Lu Y (2019). Epigenetic downregulation of MUC17 by *H. pylori* infection facilitates NF-κB-mediated expression of CEACAM1-3S in human gastric cancer. Gastric Cancer.

[9] Huang H, Wu J, Jin G, Zhang H, Ding Y, Hua Z, Zhou Y, Xue Y, Lu Y, Hu Z, Xu Y (2019). Single-nucleotide polymorphisms in Toll-like receptor genes are associated with the prognosis of gastric cancer and are not associated with *Helicobacter pylori* infection. Infect Genet Evol.

[10] Wan L, Yu W, Shen E, Sun W, Liu Y, Kong J, Wu Y, Han F, Zhang L, Yu T, Zhou Y (2019). SRSF6-regulated alternative splicing that promotes tumour progression offers a therapy target for colorectal cancer. Gut.

[11] Climente-Gonzalez H, Porta-Pardo E, Godzik A, Eyras E (2017). The functional impact of alternative splicing in cancer. Cell Rep.

[12] Yang L, He Y, Zhang Z, Wang W (2019). Systematic analysis and prediction model construction of alternative splicing events in hepatocellular carcinoma: a study on the basis of large-scale spliceseq data from The Cancer Genome Atlas. PeerJ..

[13] Xiong Y, Deng Y, Wang K, Zhou H, Zheng X, Si L, Fu Z (2018). Profiles of alternative splicing in colorectal cancer and their clinical significance: a study based on large-scale sequencing data. EBioMedicine..

[14] Wen T, Wang Z, Li Y, Li Z, Che X, Fan Y, Wang S, Qu J, Yang X, Hou K, Zhou W (2017). A four-factor immunoscore system that predicts clinical outcome for stage II/III gastric cancer. Cancer Immunol Res.

[15] Li ZX, Zheng ZQ, Wei ZH, Zhang LL, Li F, Lin L, Liu RQ, Huang XD, Lv JW, Chen FP, He XJ (2019). Comprehensive characterization of the alternative splicing landscape in head and neck squamous cell carcinoma reveals novel events associated with tumorigenesis and the immune microenvironment. Theranostics..

[16] Ryan MC, Cleland J, Kim R, Wong WC, Weinstein JN (2012). SpliceSeq: a resource for analysis and visualization of RNA-Seq data on alternative splicing and its functional impacts. Bioinformatics.

[17] Conway JR, Lex A, Gehlenborg N (2017). UpSetR: an R package for the visualization of intersecting sets and their properties. Bioinformatics.

[18] Zhou Y, Zhou B, Pache L, Chang M, Khodabakhshi AH, Tanaseichuk O, Benner C, Chanda SK (2019). Metascape provides a biologist-oriented resource for the analysis of systems-level datasets. Nat Commun.

[19] Gao J, Kwan PW, Shi D (2010). Sparse kernel learning with LASSO and Bayesian inference algorithm. Neur Netw.

[20] Camp RL, Dolled-Filhart M, Rimm DL (2004). X-tile: a new bio-informatics tool for biomarker assessment and outcome-based cut-point optimization. Clin Cancer Res.

[21] Zhang Z, Kattan MW (2017). Drawing Nomograms with R: applications to categorical outcome and survival data. Ann Transl Med.

[22] Rousson V, Zumbrunn T (2011). Decision curve analysis revisited: overall net benefit, relationships to ROC curve analysis, and application to case-control studies. BMC Med Inform Decis Mak.

[23] Balachandran VP, Gonen M, Smith JJ, DeMatteo RP (2015). Nomograms in oncology: more than meets the eye. Lancet Oncol.

[24] Lena PG, Paz-Gallardo A, Paramio JM, García-Escudero R (2017). Clusterization in head and neck squamous carcinomas based on lncRNA expression: molecular and clinical correlates. Clin Epigenet.

[25] Wilkerson MD, Hayes DN (2010). ConsensusClusterPlus: a class discovery tool with confidence assessments and item tracking. Bioinformatics.

[26] Neverov AD, Artamonova II, Nurtdinov RN, Frishman D, Gelfand MS, Mironov AA (2005). Alternative splicing and protein function. BMC Bioinformatics.

[27] Liao WT, Yu CL, Lan CC, Lee CH, Chang CH, Chang LW, You HL, Yu HS (2009). Differential effects of arsenic on cutaneous and systemic immunity: focusing on CD4+ cell apoptosis in patients with arsenic-induced Bowen’s disease. Carcinogenesis.

[28] Sveen A, Kilpinen S, Ruusulehto A, Lothe RA, Skotheim RI (2016). Aberrant RNA splicing in cancer; expression changes and driver mutations of splicing factor genes. Oncogene.

[29] Yang X, Coulombe-Huntington J, Kang S, Sheynkman GM, Hao T, Richardson A, Sun S, Yang F, Shen YA, Murray RR, Spirohn K (2016). Widespread expansion of protein interaction capabilities by alternative splicing. Cell.

[30] Siegel RL, Miller KD, Jemal A (2019). Cancer statistics, 2019. CA Cancer J Clin.

[31] Ule J, Blencowe BJ (2019). Alternative splicing regulatory networks: functions, mechanisms, and evolution. Mol Cell.

[32] Matlin AJ, Clark F, Smith CW (2005). Understanding alternative splicing: towards a cellular code. Nat Rev Mol Cell Biol.

[33] Oltean S, Bates DO (2014). Hallmarks of alternative splicing in cancer. Oncogene.

[34] Shi Y, Chen Z, Gao J, Wu S, Gao H, Feng G (2018). Transcriptome-wide analysis of alternative mRNA splicing signature in the diagnosis and prognosis of stomach adenocarcinoma. Oncol Rep.

[35] Chen B, Zhang C, Wang Z, Chen Y, Xie H, Li S, Liu X, Liu Z, Chen P (2019). Mechanistic insights into Nav1 7-dependent regulation of rat prostate cancer cell invasiveness revealed by toxin probes and proteomic analysis. FEBS J.

[36] Xiao X, Su G, Brown SN, Chen L, Ren J, Zhao P (2010). Peroxisome proliferator-activated receptors γ and α agonists stimulate cardiac glucose uptake via activation of AMP-activated protein kinase. The Journal of nutritional biochemistry..

[37] Zhao H, Wu S, Li H, Duan Q, Zhang Z, Shen Q, Wang C, Yin T (2019). ROS/KRAS/AMPK signaling contributes to gemcitabine-induced stem-like cell properties in pancreatic cancer. Molecular Therapy-Oncolytics..

[38] Mazure NM, Pouysségur J (2009). Atypical BH3-domains of BNIP3 and BNIP3L lead to autophagy in hypoxia. Autophagy..

[39] Qiu J, Keyser B, Lin ZT, Wu T (2018). Autoantibodies as potential biomarkers in breast cancer. Biosensors..

[40] Binnewies M, Roberts EW, Kersten K, Chan V, Fearon DF, Merad M, Coussens LM, Gabrilovich DI, Ostrand-Rosenberg S, Hedrick CC, Vonderheide RH (2018). Understanding the tumor immune microenvironment (TIME) for effective therapy. Nat Med.

[41] Yao J, Caballero OL, Huang Y, Lin C, Rimoldi D, Behren A, Cebon JS, Hung MC, Weinstein JN, Strausberg RL, Zhao Q (2016). Altered expression and splicing of ESRP1 in malignant melanoma correlates with epithelial–mesenchymal status and tumor-associated immune cytolytic activity. Cancer Immunol Res.

[42] Zhang SC, Hu ZQ, Long JH, Zhu GM, Wang Y, Jia Y, Zhou J, Ouyang Y, Zeng Z (2019). Clinical Implications of Tumor-Infiltrating Immune Cells in Breast Cancer. Journal of Cancer..

[43] Wu Z, Wang M, Liu Q, Liu Y, Zhu K, Chen L, Guo H, Li Y, Shi B (2020). Identification of gene expression profiles and immune cell infiltration signatures between low and high tumor mutation burden groups in bladder cancer. Int J Med Sci.

[44] Pan H, Lu L, Cui J, Yang Y, Wang Z, Fan X (2020). Immunological analyses reveal an immune subtype of uveal melanoma with a poor prognosis. Aging (Albany NY)..

[45] Yuan L, Xu B, Yuan P, Zhou J, Qin P, Han L, Chen G, Wang Z, Run Z, Zhao P, Gao Q (2017). Tumor-infiltrating CD4+ T cells in patients with gastric cancer. Cancer Cell Int.

[46] Zhang D, He W, Wu C, Tan Y, He Y, Xu B, Chen L, Li Q, Jiang J (2019). Scoring system for tumor-infiltrating lymphocytes and its prognostic value for gastric cancer. Front Immunol.

[47] Lynch KW (2004). Consequences of regulated pre-mRNA splicing in the immune system. Nat Rev Immunol.

[48] Kim WM, Huang YH, Gandhi A, Blumberg RS (2019). CEACAM1 structure and function in immunity and its therapeutic implications. Semin Immunol.

[49] Nakajima A, Iijima H, Neurath MF, Nagaishi T, Nieuwenhuis EE, Raychowdhury R, Glickman J, Blau DM, Russell S, Holmes KV, Blumberg RS (2002). Activation-induced expression of carcinoembryonic antigen-cell adhesion molecule 1 regulates mouse T lymphocyte function. J Immunol.

[50] Xing Q, Wang J, Zhao Q, Liao H, Xun X, Yang Z, Huang X, Bao Z (2019). Alternative splicing, spatiotemporal expression of TEP family genes in Yesso scallop (Patinopecten yessoensis) and their disparity in responses to ocean acidification. Fish Shellfish Immunol.

[51] Will CL, Lührmann R (2011). Spliceosome structure and function. Cold Spring Harbor.

[52] Warzecha CC, Shen S, Xing Y, Carstens RP (2009). The epithelial splicing factors ESRP1 and ESRP2 positively and negatively regulate diverse types of alternative splicing events. RNA Biol.

[53] Munkley J, Li L, Gokul Krishnan SR, Hysenaj G, Scott E, Dalgliesh C, Zarni HO, Maia TM, Cheung K, Ehrmann I (2019). ESRP2Androgen-regulated transcription of drives alternative splicing patterns in prostate cancer. Life.

[54] Yu M, Hong W, Ruan S, Guan R, Tu L, Huang B, Hou B, Jian Z, Ma L, Jin H (2019). Genome-wide profiling of prognostic alternative splicing pattern in pancreatic cancer. Frontiers in oncology..

[55] Cho CY, Chung SY, Lin S, Huang JS, Chen YL, Jiang SS, Cheng LC, Kuo TH, Lay JD, Yang YY, Lai GM (2019). PTBP1-mediated regulation of AXL mRNA stability plays a role in lung tumorigenesis. Sci Rep.

